# The use of T-DNA insertional mutagenesis to improve cellulase production by the thermophilic fungus *Humicola insolens* Y1

**DOI:** 10.1038/srep31108

**Published:** 2016-08-10

**Authors:** Xinxin Xu, Jinyang Li, Pengjun Shi, Wangli Ji, Bo Liu, Yuhong Zhang, Bin Yao, Yunliu Fan, Wei Zhang

**Affiliations:** 1 Biotechnology Research Institute, Chinese Academy of Agricultural Sciences, Beijing 100081, China; 2 Key Laboratory of Feed Biotechnology of the Ministry of Agriculture, Feed Research Institute, Chinese Academy of Agricultural Sciences, Beijing 100081, China

## Abstract

*Humicola insolens* is an excellent producer of pH-neutral active, thermostable cellulases that find many industrial applications. In the present study, we developed an efficient *Agrobacterium tumefaciens*-mediated transformation system for *H. insolens*. We transformed plasmids carrying the promoter of the glyceraldehyde-3-phosphate dehydrogenase gene of *H. insolens* driving the transcription of genes encoding neomycin phosphotransferase, hygromycin B phosphotransferase, and enhanced green fluorescent protein. We optimized transformation efficiency to obtain over 300 transformants/10^6^ conidia. T-DNA insertional mutagenesis was employed to generate an *H. insolens* mutant library, and we isolated a transformant termed T4 with enhanced cellulase and hemicellulase activities. The FPase, endoglucanase, cellobiohydrolase, *β*-glucosidase, and xylanase activities of T4, measured at the end of fermentation, were 60%, 440%, 320%, 41%, and 81% higher than those of the wild-type strain, respectively. We isolated the sequences flanking the T-DNA insertions and thus identified new genes potentially involved in cellulase and hemicellulase production. Our results show that it is feasible to use T-DNA insertional mutagenesis to identify novel candidate genes involved in cellulase production. This will be valuable when genetic improvement programs seeking to enhance cellulase production are planned, and will also allow us to gain a better understanding of the genetics of the thermophilic fungus *H. insolens*.

Lignocellulose is the most abundant renewable biological resource on Earth and can be converted to mixed sugars prior to fermentation to yield biofuels and many other useful biomaterials. The use of enzymes active on carbohydrates to catalyze lignocellulose degradation has long been considered to be the most promising strategy. Enzyme-catalyzed processes, in contrast to acid hydrolysis, afford high yields of fermentable sugars and minimize environmental pollution. Presently, most commercially available cellulases used for biomass degradation are derived from *Trichoderma reesei*^1^. Although many studies have used classical mutagenesis and genetic modification in efforts to obtain hypercellulolytic *T. reesei* mutants^2^,^3^, the relatively low *β*-glucosidase activity of the strain, poor hemicellulose production, the need for acidophilic culture conditions, and poor thermal stability remain major obstacles when seeking to employ *T. reesei* to efficiently hydrolyze lignocellulose, and in many other applications.

Filamentous fungi of the genus *Humicola* are excellent producers of cellulases for industrial applications^4^. *Humicola insolens* is an innocuous non-toxic fungus producing a comprehensive profile of cellulases and hemicellulases, including at least two cellobiohydrolases, seven endoglucanases, two *β*-glucosidases, five xylanases, and three xylosidases^5^,^6^,^7^,^8^,^9^,^10^,^11^,^12^,^13^. Recently, two further novel *β*-glucosidases of the glycosyl hydrolase family 3 were identified in *H. insolens* strain Y1 (unpublished data). Unlike most enzymes from acidophilic and mesophilic fungi, the cellulases and hemicellulases of *H. insolens* are active under neutral conditions, are alkali-tolerant, and exhibit good thermostability^5^,^7^,^11^. Such properties render enzymes secreted by *H. insolens* outstanding choices for applications in many industries. EGV (Carezyme; Novozymes) has dominated the laundry market for several years, and a multi-active *β*-glucanase preparation from *H. insolens* (Ultraflo L, Novozymes) is used by breweries in the mashing process. In addition, hemicellulases and cellulases from *H. insolens* degrade lignocellulose-rich materials, such as rice straw or wood chips, much more efficiently than do enzymes from *T. reesei*^14^. *H. insolens* also has served as an excellent host for overproduction of heterologous enzymes, especially neutral cellulases^15^. Thus, *H. insolens* should be seriously considered as an alternative to *T. reesei* in terms of biomass degradation.

However, despite the dramatic developments in biotechnology over the past few decades, few reports to date have focused on the genetics or molecular biology of *H. insolens*. In the present study, we establish an efficient *Agrobacterium tumefaciens*-mediated transformation (ATMT) system for *H. insolens* and create a mutant library using this system. Further, we isolate a mutant (termed T4) with enhanced cellulolytic capacity and identify the sequences flanking the T-DNA insertion sites. These results will help us gain a better understanding of the genetics of the organism and will greatly facilitate future genetic engineering of the fungus to obtain strains producing high levels of cellulase.

## Materials and Methods

### Strains, media, and growth conditions

*H. insolens* Y1^16^ was used as the recipient for transformation. Strain AGL-1 of *A. tumefaciens* was used to transform *H. insolens*.

To trigger sporulation, *H. insolens* Y1 was grown in potato-dextrose agar (PDA) medium for 7–12 days at 42 °C. The minimal medium (MM), induction medium (IM), and co-cultivation medium (CM) used for *A. tumefaciens*-mediated transformation (ATMT) were prepared as described previously^17^. MNN medium (per liter: 1 g tryptone, 20 g yeast extract, 0.6 g MgSO_4_·7H_2_O, 0.3 g CaCl_2_·2H_2_O, and 20 g Avicel) was used for fermentation. *A. tumefaciens* was routinely grown at 28 °C.

### Sensitivity testing for geneticin and hygromycin

The sensitivity of *H. insolens* to geneticin and hygromycin B was monitored by growing the strain on PDA medium. Spore suspensions (about 100 μL of a suspension of 1.0 × 10^5^ spores/mL) were spread on PDA plates supplemented with various concentrations of geneticin (0, 25, 50, 100, and 150 μg/mL) or hygromycin B (0, 5, 10, 25, and 50 μg/mL). Growth was monitored during incubation at 42 °C for 3–5 days.

### Plasmid construction

The promoter and terminator regions of the glyceraldehyde 3-phosphate dehydrogenase (*gpd*) gene were amplified with primers Pgpd-F/Pgpd-R and Tgpd-F/Tgpd-R (shown in Table 1), respectively. The PCR fragments were ligated into pEASY-Blunt (Transgen, China) to generate pB-Pgpd and pB-Tgpd, respectively. Next, pB-Tgpd was digested with *EcoR*V and *Xho*I and ligated into the corresponding sites of pBluescript I KS (+) to yield pTgpd. Next, pB-Pgpd was digested with *Not*I and *BamH*I and subcloned into the corresponding sites of pTgpd to generate pPgpd-T. A geneticin-resistance gene (*neo*) was amplified from pEGFP-N1 (Clontech) with the aid of primers npt-F/npt-R. After cloning into pEASY-Blunt, the amplified DNA fragments were digested with *BamH*I-*EcoR*V and subcloned into the corresponding sites of the vector pPgpd-T, to generate pP-neo-T. Then, the entire Pgpd-*neo*-Tgpd fragment was amplified from pP-neo-T using Pgpd-F-2/Tgpd-R. The fragment was subcloned into pEASY-Blunt, digested with *Spe*I and *Xho*I, and ligated into the corresponding sites of pAg1-H3^18^ to generate pAg1-neo, in which the *hph* gene driven by the *A. nidulans trpC* promoter was thus replaced by the *neo* gene driven by the *H. insolens gpd* promoter. We also constructed a plasmid that could be used to express heterogeneous genes. The strategy was as follows: First, pAg1-H3 was digested with *Bgl*II and self-ligated to remove the PtrpC-*hph*-TtrpC cassette, yielding pAg1. Next, the *hph* fragment was amplified from pAg1-H3 using the primers hph-F/hph-R. After ligation into pEASY-Blunt, the *hph* fragment was digested with *BamH*I and *EcoR*V and subcloned into the corresponding sites of the pPgpd-T-2 vector. This vector was constructed in the same manner as pPgpd-T except that Pgpd was amplified with Pgpd-F-3/Pgpd-R to remove the *Sma*I site located upstream of Pgpd-F-3, to yield pP-hph-T. The entire Pgpd-*hph*-Tgpd cassette was obtained by *Pvu*II digestion and ligated into the corresponding site of pAg1 to generate pAg1-hyg. Next, Pgpd and Tgpd fragments were obtained from pPgpd-T-2 via *Pvu*II digestion and ligated into the *Swa*I site of pAg1-hyg, to yield pAg1-hyg-P-T. As only one *Sma*I site lies between Pgpd and Tgpd [the site is derived from pBluescript I KS(+)] in pAg1-hyg-P-T, any gene can be blunt-end ligated into that site. The *egfp* gene was amplified from pEGFP-N1 (Clontech) using primers gfp-F/gfp-R, and ligated into pEASY-Blunt. After digestion with *EcoR*V, the fragment was cloned into the *Sma*I site of pAg1-hyg-P-T, in the appropriate direction, to yield pAg-egfp. Plasmids pAg1-neo and pAg-egfp were introduced into wild-type *H. insolens* by ATMT.

### *A. tumefaciens*-mediated transformation

The transformation procedure was based on a previously described protocol^19^ with some modifications. Spores were obtained on PDA medium after culture at 42 °C for 10 days. *A. tumefaciens* was grown at 28 °C for 2 days in liquid MM medium supplemented with 50 μg/mL kanamycin. Bacterial cell suspensions were subsequently diluted to an optical density at 600 nm (*OD*_600_) of 0.2; induction medium (IM) with 200–500 μM acetosyringone (AS) was used for dilution. The cells were grown for an additional 6 h to an *OD*_600_ of 0.4–0.8. Next, 100 μL of bacterial culture previously induced with 400 μM AS were added to CM plates containing fungal mycelia that had been allowed to germinate for various periods (6–30 h) on cellophane paper. Co-cultivation proceeded at various temperatures (22 °C, 25 °C, or 28 °C) for 24–96 h. Next, the mixed cultures were transferred to PDA medium supplemented with 200 μg/mL cefotaxime and 100 μg/mL geneticin to select fungal transformants. Each factor was varied with all other factors held constant.

### Fluorescence microscopy

The hyphae of randomly selected *H. insolens* transformant harboring pAg*-*egfp were prepared on PDA plates without hygromycin B. After 2 d growth, the edge of the colony was observed. The green fluorescence emission from EGFP was detected using a Nikon Eclipse Ni microscope (Nikon, Japan). Images were taken under 40 × objective and processed with NIS-Elements BR 3.0 imaging software (Nikon, Japan).

### Molecular analysis of transformants

Fungal genomic DNA was extracted from mycelia grown on PDA plates. The mycelia were harvested, dried with filter paper, and ground in liquid nitrogen using a sterilized mortar and pestle. Genomic DNA was isolated with the aid of a DNA Quick Plant System (TianGen, China). The *neo* gene was detected via PCR using primers neo-F/neo-R, which amplified a 555-bp sequence spanning the gene. Routine PCR amplification consisted of initial denaturation at 95 °C; followed by 30 cycles of amplification (95 °C for 30 s, 58 °C for 30 s, and 72 °C for 40 s); followed by an additional 10 min at 72 °C.

### Mitotic stability of transformants

To evaluate stability, 20 randomly selected transformants were cultured on PDA plates without geneticin for 7 d. Mycelia from the edges of the cultures were transferred to fresh PDA plates and grown for another 7 d. After repeating this procedure 5 times, germinating mycelia from each transformant were transferred to PDA plates containing 100 μg/mL geneticin.

### Screening for mutants with improved cellulase activity

T-DNA inserted mutants and the wild-type *H. insolens* Y1 strain were inoculated onto screening plates (15 g wheat bran, 0.8 g bean pulp and 0.8 g grass meal pellets were boiled in 1 L ddH_2_O for 10 min, the supernatant was harvested and the volume adjusted to 1 L with ddH_2_O, followed by addition of 2% Avicel and 1.5% agar). After growth at 42 °C for 3–5 days, the target mutants were selected on the basis of colony size and hyphal morphology using the wild-type strain as the control.

### TAIL-PCR analysis of integration junctions

TAIL-PCR was performed as previously described^20^. Genomic DNA from selected transformants was extracted and purified as described above. The degenerate primers (LAD1-1, LAD1-3, LAD1-5, and LAD1-11) and the nested LB-specific primers (LB1, LB2, and LB3) and RB-specific primers (RB-A, RB-B, and RB-C) used are listed in Table 1. Reaction products were recovered and sequenced.

### Cellulase assay

The wild-type and T4 strains were fermented in 40 mL of MNN medium inoculated with 1.0 × 10^6^ 12-day-old spores at 42 °C with shaking at 200 rpm. Cellulase activities in supernatants were assayed at various intervals. FPase, CMCase, and xylanase were assayed using the 3,5-dinitrosalicylic acid (DNS) method^21^. Whatman No.1 filter papers (1 × 6 cm in area) were immersed in 2 mL of appropriately diluted supernatants rendered to 100 mM in Na_2_HPO_4_-citric acid (pH 6.0) at 60 °C for 30 min. Next, 3 mL of DNS were added to terminate the reaction. The mixtures were boiled for 5 min and the absorbances at 540 nm determined. The standard assays for CMCase and xylanase featured the addition of 100 μL of appropriately diluted supernatants to 900 μL of 100 mM Na_2_HPO_4_-citric acid (pH 6.0) containing 1.0% (w/v) CMC-Na or birchwood xylan, followed by incubation at 60 °C for 10 min. Each reaction was terminated by the addition of 1.5 mL of DNS reagent and boiling for 5 min. The absorbance at 540 nm was determined when the reaction mixture had cooled to room temperature. Glucosidase activity was measured using *p*-nitrophenyl-*β*-D-glucopyranoside (*p*NPG) as a substrate. The reaction featured the addition of 250 μL of 4 mM *p*NPG in 100 mM Na_2_HPO_4_-citric acid (pH 6.0) to 250 μL of appropriately diluted solutions, followed by incubation at 60 °C for 10 min. Finally, 1.5 mL of 1.0 M Na_2_CO_3_ were added to terminate the reaction, and the liberated *p*-nitrophenyl was detected by measuring the absorbance at 420 nm. Cellobiohydrolase activity was measured in the same way, except that *p*-nitrophenyl-*β*-D-cellobioside (*p*NPC) served as a substrate. One unit of enzyme activity was defined as the amount of enzyme required to release 1 μmoL of reduced sugar (for FPase, CMCase, and xylanase) or 1 μmoL of *p*-nitrophenyl (for glucosidase and cellobiohydrolase) from the substrate, per min, under the conditions described above. Glucose and *p*-nitrophenyl served as standards.

### SDS-PAGE and protein assay

Sodium dodecyl sulfate (SDS)-polyacrylamide gel electrophoresis (PAGE) was performed as described by Laemmli^22^ with 12% polyacrylamide gel. Proteins were stained with Coomassie Brilliant Blue G-250. Protein concentration was measured by the Bradford method^23^ using a protein assay kit (Bio-Rad).

### Nucleotide sequence accession numbers

The nucleotide sequences of *gpd, proA* and *tdiD* from *H. insolens* Y1 have been deposited in GenBank under accession nos. KU847960, KU836630 and KU836631, respectively.

## Results

### Construction of binary vectors for *A. tumefaciens*-mediated transformation of *H. insolens* Y1

To aid in the development of a rapid and efficient transformation method for *H. insolens* Y1, we tested geneticin and hygromycin B currently used as selectable markers in fungal transformations. We found that geneticin at ≥50 μg/mL or hygromycin B at ≥10 μg/mL completely inhibited conidial germination by the *H. insolens* Y1 wild-type strain (Figure S1). Thus, both antibiotics were used to select *H. insolens* transformants.

**Figure 1:**
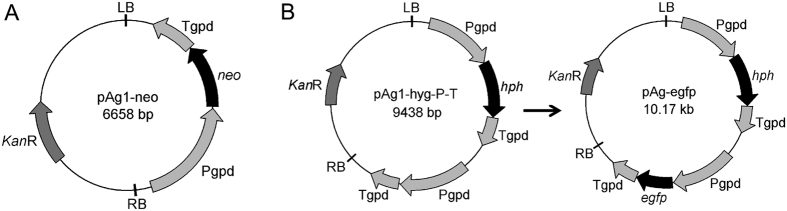
Schematic representation of pAg1-neo (**A**) and pAg-egfp (**B**). The positions of Pgpd and Tgpd (the promoter and terminator of the *H. insolens* Y1 *gpd* gene, respectively), *neo* (geneticin-resistance gene), *hph* (hygromycin B-resistance gene); *egfp* (enhanced green fluorescent protein gene), and RB and LB (the right and left borders of T-DNA, respectively) are shown.

To transform *H. insolens* Y1, we constructed the binary vector pAg1-neo using the backbone of plasmid pAg1-H3 (Fig. 1A). To efficiently drive the expression of the antibiotic-resistance gene, we cloned the promoter and terminator of the glyceraldehyde 3-phosphate dehydrogenase gene (*gpd*) from *H. insolens* and used this promoter to drive the *neo* gene. We also constructed the binary vector pAg1-hyg-P-T for expression of heterologous genes (Fig. 1B). To determine the utility of this plasmid, the gene encoding enhanced green fluorescent protein (*egfp*) was cloned into the vector, yielding pAg*-*egfp.

### Transformation competency of *H. insolens* with the constructed vectors

**Figure 2:**
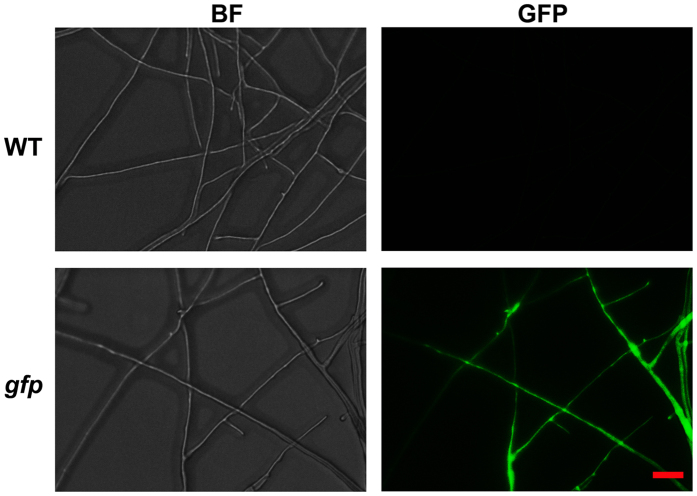
Fluorescence of mycelia of *H. insolens* transformed with the *egfp* gene. One transformant (*gfp*) was randomly selected. Hyphae were observed by both bright-field (BF) microscopy and fluorescence (GFP) microscopy after growth on PDA plates for 2 days. Bar: 10 μm.

*A. tumefaciens* strain AGL-1, harboring the binary vector pAg1-neo, was used to transform *H. insolens* Y1. Plasmid pAg1-neo was confirmed to be transformation-competent; the transformants grew on PDA plates containing 100 μg/mL geneticin (data not shown). After transformation of pAg*-*egfp into the wild-type strain, strong green fluorescence was observed by fluorescence microscopy (Fig. 2), suggesting that the *egfp* gene was successfully expressed.

### Optimization of an ATMT system for *H. insolens*

**Figure 3:**
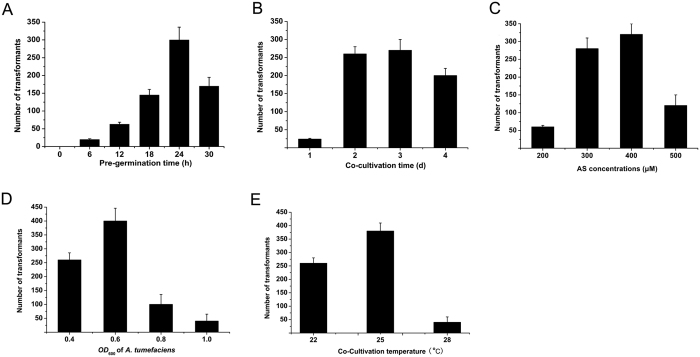
Factors affecting the transformation efficiency of *H. insolens*. (**A**) Effect of pre-germination on transformation efficiency. 400 μM AS, *OD*_600_ of 0.6 (*A. tumefaciens* cells), 25 °C (co-cultivation temperature) and 2 days (co-cultivation time) were used in the experiments. Colonies that grew were considered to be transformants. (**B**) Effect of co-cultivation time on transformation efficiency. 400 μM AS, 24 h (pre-germination time), *OD*_600_ of 0.6 (*A. tumefaciens* cells) and 25 °C (co-cultivation temperature) were used in the experiments. (**C**) Effect of AS concentration on transformation efficiency. 24 h (pre-germination time), *OD*_600_ of 0.6 (*A. tumefaciens* cells), 25 °C (co-cultivation temperature) and 2 days (co-cultivation time) were used in the experiments. (**D**) Effect of *A. tumefaciens* cell concentration on transformation efficiency. 400 μM AS, 24 h (pre-germination time), 25 °C (co-cultivation temperature) and 2 days (co-cultivation time) were used in the experiments. (**E**) Effect of co-cultivation temperature on transformation efficiency. 400 μM AS, 24 h (pre-germination time), *OD*_600_ of 0.6 (*A. tumefaciens* cells) and 2 days (co-cultivation time) were used in the experiments. Error bars represent standard deviations from three independent experiments.

The ATMT procedure for *H. insolens* was optimized by determining the effects of various parameters on transformation efficiency. Pre-germination is crucial for successful transformation. No transformants were obtained using non-germinated spores. Commencing at 6 h of pre-germination, transformation efficiency increased to 24 h, and fell thereafter (Fig. 3A). Co-cultivation time also affected transformation efficiency. Maximum efficiency was observed after 48 h of co-cultivation and was maintained to 72 h (Fig. 3B). The optimal level of AS was found to be 300–400 μM (Fig. 3C). *A. tumefaciens* cell concentration notably affected the transformation efficiency of *H. insolens*; the maximum transformation efficiency was observed at an *OD*_600_ of 0.6 (Fig. 3D). As the cell concentration increased, the transformation efficiency fell significantly. The optimal temperature for co-cultivation was 25 °C (Fig. 3E).

### Molecular analysis of transformants

**Figure 4:**
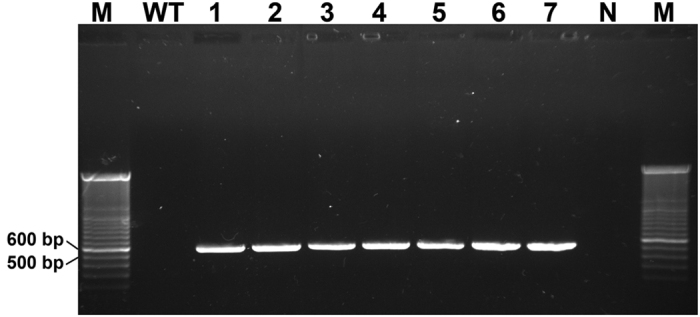
PCR products of *neo* from the genomic DNA of *H. insolens* transformants. All strains were grown on PDA plates for 2 days and genomic DNA extracted. Primers neo-F/neo-R were used. M, 100 bp DNA ladder; WT, the wild-type strain; 1–7, randomly selected transformants; N, negative control.

To confirm that T-DNA had become integrated into the *H. insolens* genome, seven putative geneticin-resistant transformants were randomly selected for PCR analysis using the *neo-*specific primers neo-F and neo-R. In all mutants, a 555-bp *neo-*specific fragment was amplified, indicating that the T-DNA was in fact integrated (Fig. 4).

### Mitotic stability of transformants

In other fungi, DNA integrated via ATMT was relatively stable during growth in the absence of selective antibiotics^24^,^25^. This is an important feature of an effective mutagenesis system. After five subcultures in the absence of geneticin, all randomly selected *H. insolens* transformants tested grew on PDA with 100 μg/mL geneticin, confirming the genetic stability of integrated DNA (Figure S2).

### T-DNA insertional mutagenesis and screening for mutants with improved cellulase activity

**Figure 5:**
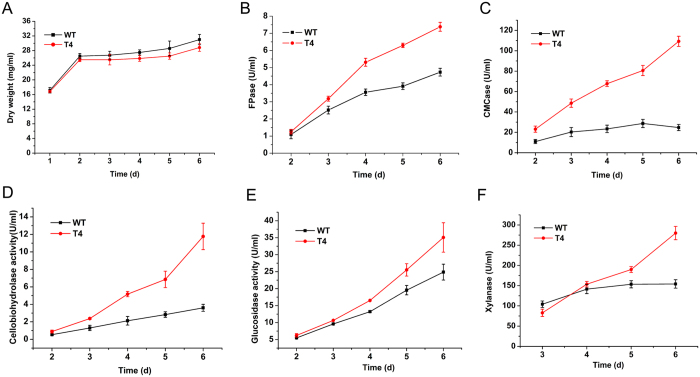
Characterization of strain T4. Over a 6-day fermentation period (**A**) dry weight, (**B**) total cellulase activity (FPase), (**C**) endoglucanase activity (CMCase), (**D**) cellobiohydrolase activity, (**E**) xylanase activity, and (**F**) glucosidase activity were measured. Error bars represent standard deviations from three independent experiments.

A T-DNA tagged mutant library of *H. insolens* was obtained by one-time ATMT transformation of about 1 × 10^7^ fungal spores. 1000 randomly selected transformants were screened by screening plates. Transformants exhibiting phenotypic changes on screening plates were fermented in MNN medium for 5 d and the cellulase activity of the supernatants was determined. One promising strain (designated T4) with obvious phenotypic changes compared to the WT (Figure S3), exhibited improved cellulolytic activity (FPase activity; 50–60% increase) when grown in fermentation medium for 4–6 days, compared to the parental strain Y1 (Fig. 5B). The endoglucanase and cellobiohydrolase activities of T4 were 4.4- and 3.2-fold higher, respectively, than those of the wild-type strain after 6 days of fermentation (Fig. 5C,D). The T4 glucosidase and xylanase activities were also elevated (by 41% and 81% at 6 days of growth, respectively) (Fig. 5E,F). T4 was further characterized in terms of the protein expression profile. SDS–PAGE showed that the T4 fermentation supernatant contained more secreted proteins than did the wild-type supernatant (Figure S4A). As the biomasses attained by the T4 and wild-type strains did not differ significantly (Fig. 5A), the enhanced cellulolytic capacity of T4 may be attributable to the secretion of more cellulolytic enzymes by that strain (compared to the wild-type strain; Figure S4B).

### Cloning of genomic DNA flanking T-DNA insertion sites

TAIL-PCR was used to isolate T4 genomic DNA segments adjacent to the T-DNA inserts. Sequencing of the flanking T-DNA junctions showed that the T4 genome had two T-DNA integration sites. One was in the promoter region (85 bp upstream of the starting ATG) of a gene encoding a putative Zn(2)-Cys(6) transcriptional regulatory protein, designated *proA*. The ProA polypeptide shares 67% sequence identity with *Cryphonectria parasitica* Pro1 and *Sordaria macrospora* Pro1, and 44% with *Aspergillus nidulans* NosA, which have previously been shown to play important roles in sexual reproduction^26^,^27^,^28^. The other integration site was in the middle of a putative aminotransferase-encoding gene, designated *tdiD*. The TdiD polypeptide shares 65% sequence identity with *Aspergillus nidulans* TdiD, which is involved in biosynthesis of the antitumor fungal metabolite terrequinone A^29^.

## Discussion

*H. insolens* produces a variety of cellulases and hemicellulases that find ready applications in industry. Nevertheless, the fungus remains poorly known genetically. Development of efficient transformation and expression systems will facilitate molecular genetic analysis and gene manipulation, including heterologous gene expression, functional analysis of targeted genes, and genetic engineering of the original strain.

ATMT is an essential tool when studying the functional genomics of filamentous fungi and has been reported to be applicable to many such fungi^18^,^19^. We describe successful *A. tumefaciens*-mediated transformation of *H. insolens*. The promoter of the endogenous glyceraldehyde 3-phosphate dehydrogenase gene efficiently drove the expression of exogenous genes, including geneticin- and hygromycin B-resistance genes and the gene encoding the enhanced green fluorescent protein. We thus constructed and tested a doubly functional binary *Agrobacterium* vector, pAg1-hyg-P-T, bearing a hygromycin B-resistance expression cassette and another copy of both Pgpd and Tgpd. pAg1-hyg-P-T can be used to express any gene of interest in *H. insolens* Y1. The plasmid can serve as an expression vector or as a backbone plasmid if gene knockout is planned. We optimized the transformation efficiency to obtain over 300 transformants/10^6^ conidia. This efficiency is similar with those obtained for *Fusarium oxysporum*^19^ (300–500 transformants/10^6^ conidia), *Aspergillus terreus* (350 transformants/10^6^ conidia)^25^ and *A. awamori* (200–250 transformants/10^6^ conidia)^30^, and much higher than many other fungi, such as *A. fumigatus* (100 transformants/10^7^ conidia)^31^, *Guignardia citricarpa* (14–16 transformants/10^6^ spores)^32^, and *Lecanicillium lecanii* (25 transformants/10^6^ conidia)^33^. This will facilitate functional genetic analysis of and enhancement of cellulase synthesis by *H. insolens*.

In addition to the introduction of foreign genes, ATMT has also been an effective way for strain improvement, since non-homologous recombination of T-DNA into the host genome often triggers gene disruption, creating mutant phenotypes^34^. ATMT has been applied to eliminate mycotoxin production by an industrially important strain of *Monascus purpureus*^35^, to enhance the resistance of *Lecanicillium lecanii* to benzimidazole fungicides^33^, and to improve cellulase production by *T. reesei*^36^. Our T4 mutant exhibited a cellulolytic capacity that was dramatically greater than that of the wild-type. The FPase activity increased by 60%, attaining a level equal to that obtained when a multistep mutation strategy (alternating ultraviolet light and chemical treatments) was employed^37^. In particular, the endoglucanase and cellobiohydrolase activities of T4 were 4.4- and 3.2-fold higher than those of the wild-type strain, respectively. These figures were much higher than those obtained using a multistep mutation strategy^37^. Moreover, the glucosidase and xylanase activities also improved, by 41% and 81%, respectively, suggesting that T-DNA insertional mutagenesis globally enhanced cellulase and hemicellulase production. These results showed that ATMT effectively improved cellulase production by *H. insolens*.

The pivotal advantage of insertional mutagenesis compared to chemical or radiation mutagenesis is that the disrupted genes and their flanking sequences can be conveniently identified^38^. In the present study, we identified T-DNA insertions in two genes, *proA* and *tdiD*, from the cellulase-hyperproducing mutant T4. *tdiD* encodes a protein of the pyridoxal phosphate (PLP)-dependent aspartate aminotransferase superfamily, which participates in the secondary metabolism of *Aspergillus nidulans*^29^. *proA* encodes a putative transcription factor that contains a GAL4-like Zn(II)2Cys6-binuclear cluster DNA-binding domain and a fungal-specific transcription factor domain. Proteins homologous to ProA play important roles in many fungal biological processes. The first identified Pro1 (that of *S. macrospora*) plays a central role in sexual development^26^,^39^. In *Alternaria brassicicola*, disruption of *pro1* resulted in significant reductions in virulence and the rate of vegetative growth^40^. ProA is an essential regulator of the mutual symbiotic interaction between *Epichloë festucae* and perennial ryegrass^28^. However, no study has yet found that ProA-like proteins function in cellulase production. Our results suggest that *proA* and/or *tdiD* are novel candidate genes that may be involved in cellulase and hemicellulase production by *H. insolens*, even though their contribution to these processes needs to be validated by further studies.

## Additional Information

**How to cite this article**: Xu, X. *et al*. The use of T-DNA insertional mutagenesis to improve cellulase production by the thermophilic fungus *Humicola insolens* Y1. *Sci. Rep.* **6**, 31108; doi: 10.1038/srep31108 (2016).

## Supplementary Material

Supplementary Information 1

Supplementary Information 2

## Figures and Tables

**Table 1:** Primers used in this study.

Primer	Sequence (5′-3′)^a^
Pgpd-F	GCGGCCGCCAGTGCTGCACATACAGAG
Pgpd-F-2	ACTAGTCCAGTGCTGCACATACAGAG
Pgpd-F-3	TGCGGCCGCATGTCAGGAACCTCAAC
Pgpd-R	GGATCCTGGCTGTGAGATGGGAGTGAG
Tgpd-F	GATATCGTGCAAATTTATAGGCGGC
Tgpd-R	CTCGAGACCAACCCAACCTCCACCC
npt-F	GGATCCATGATTGAACAAGATGGA
npt-R	GATATCTCAGAAGAACTCGTCAAGAAG
neo-F	CAACAGACAATCGGCTGC
neo-R	GCCACAGTCGATGAATCC
hph-F	CGGATCCATGAAAAAGCCTGAAC
hph-R	CGATATCCTATTCCTTTGCCCTCGG
gfp-F	GATATCATGGTGAGCAAGGGCGAG
gfp-R	GATATCACTTGTACAGCTCGTCCATG
LAD1-1	ACGATGGACTCCAGAVNVNNNGGAA
LAD1-3	ACGATGGACTCCAGAVVNVNNNCCAA
LAD1-5	ACGATGGACTCCAGAVNVNNNTCAG
LAD1-11	ACGATGGACTCCAGABNBNNNGCTA
AC1	ACGATGGACTCCAGAG
LB-1	GGAGGTTGGGTTGGTCTCGAGATC
LB-2	ACGATGGACTCCAGTCCGGCCGTACCGAGCTCGAATTCAC
LB-3	GTTGCGCAGCCTGAATGGCGAATG
RB-A	GTTGAGGTTCCTGACATGCC
RB-B	ACGATGGACTCCAGGTAGTAGAAGTATGTACCTCTG
RB-C	GCATGCAAGCTTCGTGACTCCC

^a^The underlined nucleotide sequences indicate restriction enzyme sites.
