# Supplementary material for: The use of T-DNA insertional mutagenesis to improve cellulase production by the thermophilic fungus Humicola insolens Y1
Source: Sci Rep. 2016 Aug 10;6:31108. doi: 10.1038/srep31108 (PMC4979032; doi:10.1038/srep31108)
Supplement: Supplementary Information 1 [file srep31108-s1.pdf]

The use of T-DNA insertional mutagenesis to improve cellulase production by the thermophilic fungus *Humicola insolens* Y1

Xinxin Xu[1¶], Jinyang Li[1¶], Pengjun Shi[2], Wangli Ji[1], Bo Liu[1], Yuhong Zhang[1], Bin Yao[2*], Yunliu Fan[1], Wei Zhang[1*]

[1]Biotechnology Research Institute, Chinese Academy of Agricultural Sciences, Beijing 100081, China

[2]Key Laboratory of Feed Biotechnology of the Ministry of Agriculture, Feed Research Institute, Chinese Academy of Agricultural Sciences, Beijing 100081, China

[¶]These authors contributed equally to this work

*Corresponding author.

E-mail: zw_bio@caas.cn (W. Z.), binyao@caas.cn (B. Y.)

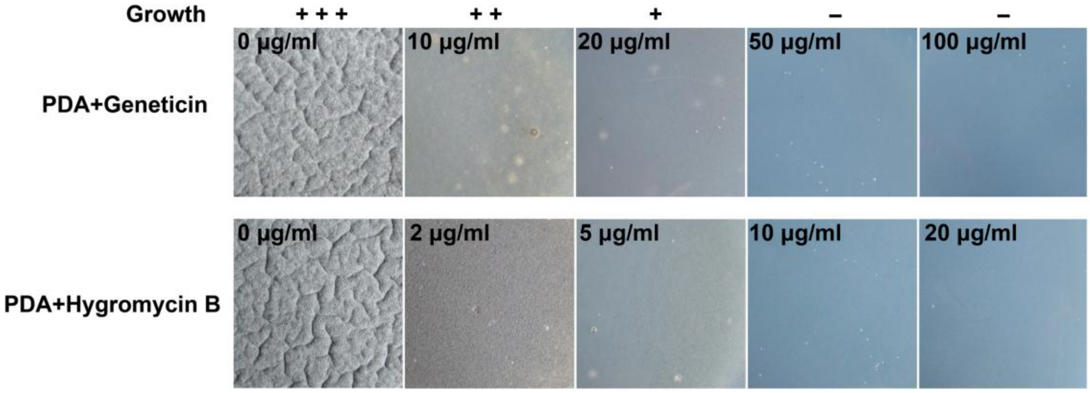

Figure S1. Effects of geneticin and hygromycin B against *Humicola insolens* Y1. +++, grow very well; ++, grow well; +, slightly grow; -, no growth

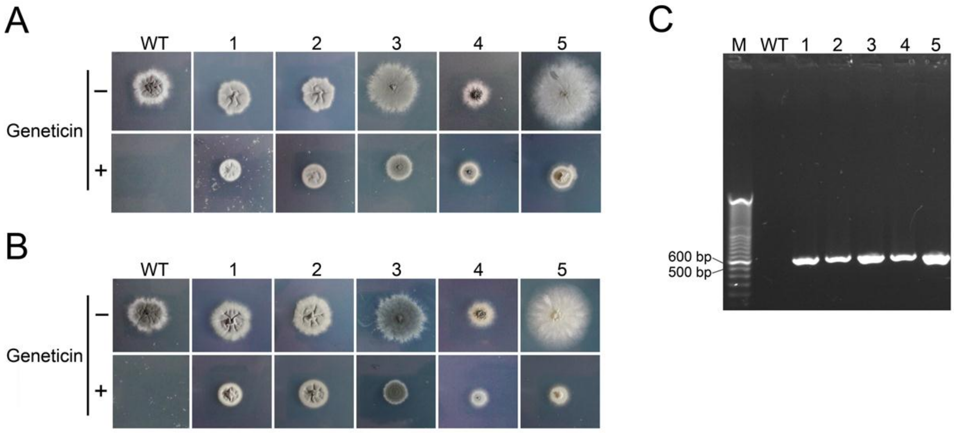

Figure S2. Mitotic stability of *H. insolens* transformants. First generation (A) and fifth generation (B) transformants grown on PDA plates with and without geneticin (100 μg/ml). (C) PCR analysis of the transformants after 5th generation on PDA plates without geneticin using primers neo-F/neo-R. WT, the wild-type strain; 1-5, randomly selected transformants harboring the plasmid pAg1-neo; M, 100 bp DNA ladder.

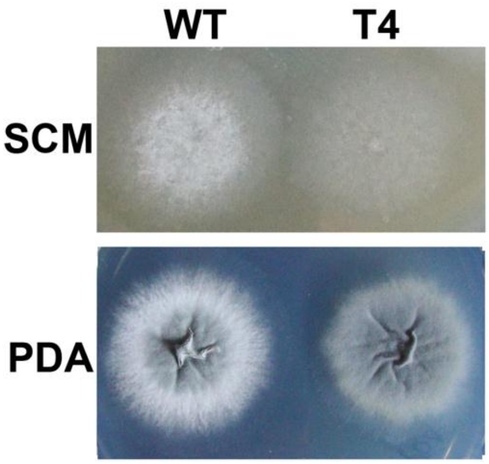

Figure S3. Growth phenotypes of the wild-type *H. insolens* (WT) and T4 grown on screening (SCM) or PDA plates.

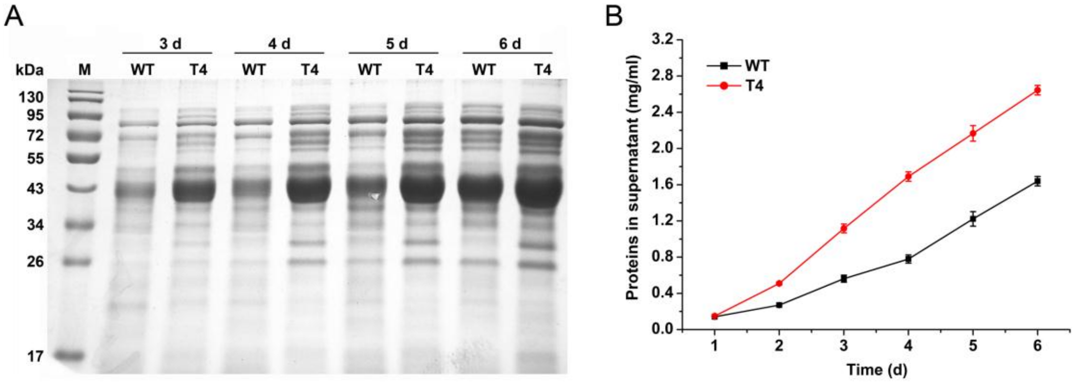

Figure S4. The protein production of WT and T4 during fermentation. (A) SDS-PAGE of supernatants of T4 and wild-type cultures grown for the indicated times. The same amount of supernatant (10 μL) was loaded into all wells. (B) The protein concentration of culture supernatants of T4 and wild-type strain. Both strains were fermented under submerged conditions in medium with 2.0% (w/v) Avicel as the carbon source at 42 ℃ with shaking at 200 rpm. Error bars represent standard deviations from three independent experiments.
