# Supplementary material for: The use of T-DNA insertional mutagenesis to improve cellulase production by the thermophilic fungus Humicola insolens Y1
Source: Sci Rep. 2016 Aug 10;6:31108. doi: 10.1038/srep31108 (PMC4979032; doi:10.1038/srep31108)
Supplement: Supplementary Information 2 [file srep31108-s2.doc]

**The use of T-DNA insertional mutagenesis to improve cellulase production by the thermophilic** **fungus *Humicola insolens* Y1**

Xinxin Xu^1¶^, Jinyang Li^1¶^, Pengjun Shi^2^, Wangli Ji^1^, Bo Liu^1^, Yuhong Zhang^1^, Bin Yao^2 *^ , Yunliu Fan^1^, Wei Zhang^1 *^

^1^Biotechnology Research Institute, Chinese Academy of Agricultural Sciences, Beijing 100081, China

^2^Key Laboratory of Feed Biotechnology of the Ministry of Agriculture, Feed Research Institute, Chinese Academy of Agricultural Sciences, Beijing 100081, China

^¶^These authors contributed equally to this work

*Corresponding author.

E-mail: zw_bio@caas.cn (W. Z.), [binyao@caas.cn](mailto:binyao@caas.cn) (B. Y.)

>pAg1-neo

CAAATTGACGCTTAGACAACTTAATAACACATTGCGGACGTTTTTAATGTACTGGGGTGGTTTTTCTTTTCACCAGTGAGACGGGCAACAGCGGCGCCATTCGCCATTCAGGCTGCGCAACTGTTGGGAAGGGCGATCGGTGCGGGCCTCTTCGCTATTACGCCAGCTGGCGAAAGGGGGATGTGCTGCAAGGCGATTAAGTTGGGTAACGCCAGGGTTTTCCCAGTCACGACGTTGTAAAACGACGGCCAGTGAATTCGAGCTCGGTACCAAGGCCCGGGCTGGCCACGGCCGCCTAGGCGCGCAAGGATCCTCTAGATCTCGAGACCAACCCAACCTCCACCCCATGTCATCCAATCATTCATCATGCCAAAGCACTCAAAGTTGTGAATGACTGCTCTCCGGGTTGTGAACGTCACAAACAAATAAAGTAGCATTGCTTGAACTAAAAATAAGTAGTTTGATCTCATCATGATCTCTTGCCGGCCTTTGCTTCATCATACCCCAAGTCTCACCAGCGTAACACCACAACATGGCTGATGTCTGGGTTGAGAGTAATACTTACACCACAAACAGGATGAATAACATCAAGAACTCCTCCAAACGCAAGAGTTTGGTGCTCAAGCGGGTCACTACAGTGGGCAAGTTATGACACTCCCAAGAACACATTCACACAAACTGGCACACACTCGCTTTGGGCTTTTCACATTTATATTGTGCTCATGGCTGCCTAACCGTGTCTCGTGTTCTCGTTTACCCTCCCATTGTTGAGATGTACCCGACCCTCTTTTCCTCAGAGTCCAAGATACTCAATTCCTCCCAATGGCTAACAAAAGCCCTCCATAAGTCACAAACTGCCGCCTATAAATTTGCACGATATCTCAGAAGAACTCGTCAAGAAGGCGATAGAAGGCGATGCGCTGCGAATCGGGAGCGGCGATACCGTAAAGCACGAGGAAGCGGTCAGCCCATTCGCCGCCAAGCTCTTCAGCAATATCACGGGTAGCCAACGCTATGTCCTGATAGCGGTCCGCCACACCCAGCCGGCCACAGTCGATGAATCCAGAAAAGCGGCCATTTTCCACCATGATATTCGGCAAGCAGGCATCGCCATGGGTCACGACGAGATCCTCGCCGTCGGGCATGCTCGCCTTGAGCCTGGCGAACAGTTCGGCTGGCGCGAGCCCCTGATGCTCTTCGTCCAGATCATCCTGATCGACAAGACCGGCTTCCATCCGAGTACGTGCTCGCTCGATGCGATGTTTCGCTTGGTGGTCGAATGGGCAGGTAGCCGGATCAAGCGTATGCAGCCGCCGCATTGCATCAGCCATGATGGATACTTTCTCGGCAGGAGCAAGGTGAGATGACAGGAGATCCTGCCCCGGCACTTCGCCCAATAGCAGCCAGTCCCTTCCCGCTTCAGTGACAACGTCGAGCACAGCTGCGCAAGGAACGCCCGTCGTGGCCAGCCACGATAGCCGCGCTGCCTCGTCTTGCAGTTCATTCAGGGCACCGGACAGGTCGGTCTTGACAAAAAGAACCGGGCGCCCCTGCGCTGACAGCCGGAACACGGCGGCATCAGAGCAGCCGATTGTCTGTTGTGCCCAGTCATAGCCGAATAGCCTCTCCACCCAAGCGGCCGGAGAACCTGCGTGCAATCCATCTTGTTCAATCATGGATCCTGGCTGTGAGATGGGAGTGAGTAGACTGCTTGTCGAGAAGAAAGGATGACGACGATGACACAGGACAGGACGTGGGGGTTGGAGAGCACACGGAGGTATGGATGATGGGGGAAGCGGATGGATGGCAGGCGGGGTACTGAAGGGGTTTTTAAGCTTCCAGCCAGGTCGGTCGGTCGTCTGAGACAGGACAGTCCGGGACCGGCAGTGGGCAGCTCAGCAAACGCGAGAGGAGTGTGCTGGTTCGCGTAGGCTCCGATACAGGCGAGATGAGGCAGTGCGGGGCCACCAGGTAGGTGCTTGCGAAACGAAGGTTGGGGGAGCTCAGGGTACCTTTGAGGAAGCTAGAGGAAGCTTGCTTACCTGAGGGATGGATGGGATCTGGATGTGAAATTGGAGGGTCAAGTGGTGAGCTGTTGGCAATGGAAGAGAATTGAGCGAGTCTGGGTCGAGTGGTTTGTGGGATGATGGAGAGAAAAAGAGGGTTTGCTTCCAACTTTGTTTCGTCTTATGGTGGCATGAGCGACAATAAAAATGCTTGACCCCTCCAAGCTGGAAGGCGGGGTGGTGCTTCTCTCGGCCGCACTTGCATCACAAATGGCGCTGGGGTGGGGTCATGTCGACGACCTTCCGTCCCTTTCGTCTCCGTTTTGGCATCTGTCGTTAGTGGGCGACTTCCGCGGCGTGCCTTCCCATGGCCACTCCTGACGGCGTGGGGGGGTCAATTTTCAATGGCTTCCTCCCAATTAACTGTCAGCAATTGCATCACAAATCCATTGTCACCGTGTATCTTCATAGCCCCCTTCCGTGGGCGCGCCCTGTCGAGACTCGCCACGGCCGCCGGACCTCCGTCACCGGGCGACACTCGGCGACGCCAGCTCCCTGATCGGGCGGCCTGCTTTCATAAGCAGCCCATTTCCAAGCTTTGTCCGGTCTTGGCCTGTCCGACCTGCGTGGCCTGTTCGTTGTGTGAAAGACCGCAATGCAAAGATCACTTTGGCTCTCAGGTAGGTTCGTATTGCGGCGTGCCATCGCCACCAGTTCAACCAACCGGTGCGGGCAGCTTGCCGCTTACCTGAAAGTCATTACCTGCTGTTTGAATTGACTACGGCTTTGTTTCGCAAGGCCACCACTAACCTCGGAAGTCTGCGATGGACCACGAAAATGATCGTGGATGAGCGGCAATGGAAAAGAACGGGTTCCGGCTAGGGACGGAGGCGCTTCACGATAATCTCGGCCCAAGCTCGACGCACTCCGGCCGTTGAGGTTCCTGACATGCCCGGGTCTCGGCCGGGTCCCAGCGCAACGGCCATATTCCTGTCTGTAGTAGAAGTATGTACCTCTGTATGTGCAGCACTGGACTAGTGCGCGATCGCGGCCGGCCGGCGCGCCGTTTAAACGGATTTAAATTAATTAATGTCGACCTGCAGGCATGCAAGCTTCGTGACTCCCTTAATTCTCCGCTCATGATCAGATTGTCGTTTCCCGCCTTCAGTTTAAACTATCAGTGTTTGACAGGATATATTGGCGGGTAAACCTAAGAGAAAAGAGCGTTTATTAGAATAATCGGATATTTAAAAGGGCGTGAAAAGGTTTATCCGTTCGTCCATTTGTTTGTTCATGCCAACCACAGGGTTCCAGATCCGACGAGCAAGGCAAGACCGAGCGCCTTTGCGACGCTCACCGGGCTGGTTGCCCTCGCCGCTGGGCTGGCGGCCGTCTATGGCCCTGCAAACGCGCCAGAAACGCCGTCGAAGCCGTGTGCGAGACACCGCGGCCGCCGGCGTTGTGGATACCTCGCGGAAAACTTGGCCCTCACTGACAGATGAGGGGCGGACGTTGACACTTGAGGGGCCGACTCACCCGGCGCGGCGTTGACAGATGAGGGGCAGGCTCGATTTCGGCCGGCGACGTGGAGCTGGCCAGCCTCGCAAATCGGCGAAAACGCCTGATTTTACGCGAGTTTCCCACAGATGATGTGGACAAGCCTGGGGATAAGTGCCCTGCGGTATTGACACTTGAGGGGCGCGACTACTGACAGATGAGGGGCGCGATCCTTGACACTTGAGGGGCAGAGTGCTGACAGATGAGGGGCGCACCTATTGACATTTGAGGGGCTGTCCACAGGCAGAAAATCCAGCATTTGCAAGGGTTTCCGCCCGTTTTTCGGCCACCGCTAACCTGTCTTTTAACCTGCTTTTAAACCAATATTTATAAACCTTGTTTTTAACCAGGGCTGCGCCCTGTGCGCGTGACCGCGCACGCCGAAGGGGGGTGCCCCCCCTTCTCGAACCCTCCCGGCCCGCTAACGCGGGCCTCCCATCCCCCCAGGCGTACGCCACTGGAGCACCTCAAAAACACCATCATACACTAAATCAGTAAGTTGGCAGCATCACCCATAATTGTGGTTTCAAAATCGGCTCCGTCGATACTATGTTATACGCCAACTTTGAAAACAACTTTGAAAAAGCTGTTTTCTGGTATTTAAGGTTTTAGAATGCAAGGAACAGTGAATTGGAGTTCGTCTTGTTATAATTAGCTTCTTGGGGTATCTTTAAATACTGTAGAAAAGAGGAAGGAAATAATAAATGGCTAAAATGAGAATATCACCGGAATTGAAAAAACTGATCGAAAAATACCGCTGCGTAAAAGATACGGAAGGAATGTCTCCTGCTAAGGTATATAAGCTGGTGGGAGAAAATGAAAACCTATATTTAAAAATGACGGACAGCCGGTATAAAGGGACCACCTATGATGTGGAACGGGAAAAGGACATGATGCTATGGCTGGAAGGAAAGCTGCCTGTTCCAAAGGTCCTGCACTTTGAACGGCATGATGGCTGGAGCAATCTGCTCATGAGTGAGGCCGATGGCGTCCTTTGCTCGGAAGAGTATGAAGATGAACAAAGCCCTGAAAAGATTATCGAGCTGTATGCGGAGTGCATCAGGCTCTTTCACTCCATCGACATATCGGATTGTCCCTATACGAATAGCTTAGACAGCCGCTTAGCCGAATTGGATTACTTACTGAATAACGATCTGGCCGATGTGGATTGCGAAAACTGGGAAGAAGACACTCCATTTAAAGATCCGCGCGAGCTGTATGATTTTTTAAAGACGGAAAAGCCCGAAGAGGAACTTGTCTTTTCCCACGGCGACCTGGGAGACAGCAACATCTTTGTGAAAGATGGCAAAGTAAGTGGCTTTATTGATCTTGGGAGAAGCGGCAGGGCGGACAAGTGGTATGACATTGCCTTCTGCGTCCGGTCGATCAGGGAGGATATCGGGGAAGAACAGTATGTCGAGCTATTTTTTGACTTACTGGGGATCAAGCCTGATTGGGAGAAAATAAAATATTATATTTTACTGGATGAATTGTTTTAGTACCTAGATGTGGCGCAACGATGCCGGCGACAAGCAGGAGCGCACCGACTTCTTCCGCATCAAGTGTTTTGGCTCTCAGGCCGAGGCCCACGGCAAGTATTTGGGCAAGGGGTCGCTGGTATTCGTGCAGGGCAAGATTCGGAATACCAAGTACGAGAAGGACGGCCAGACGGTCTACGGGACCGACTTCATTGCCGATAAGGTGGATTATCTGGACACCAAGGCACCAGGCGGGTCAAATCAGGAATAAGGGCACATTGCCCCGGCGTGAGTCGGGGCAATCCCGCAAGGAGGGTGAATGAATCGGACGTTTGACCGGAAGGCATACAGGCAAGAACTGATCGACGCGGGGTTTTCCGCCGAGGATGCCGAAACCATCGCAAGCCGCACCGTCATGCGTGCGCCCCGCGAAACCTTCCAGTCCGTCGGCTCGATGGTCCAGCAAGCTACGGCCAAGATCGAGCGCGACAGCGTGCAACTGGCTCCCCCTGCCCTGCCCGCGCCATCGGCCGCCGTGGAGCGTTCGCGTCGTCTCGAACAGGAGGCGGCAGGTTTGGCGAAGTCGATGACCATCGACACGCGAGGAACTATGACGACCAAGAAGCGAAAAACCGCCGGCGAGGACCTGGCAAAACAGGTCAGCGAGGCCAAGCAGGCCGCGTTGCTGAAACACACGAAGCAGCAGATCAAGGAAATGCAGCTTTCCTTGTTCGATATTGCGCCGTGGCCGGACACGATGCGAGCGATGCCAAACGACACGGCCCGCTCTGCCCTGTTCACCACGCGCAACAAGAAAATCCCGCGCGAGGCGCTGCAAAACAAGGTCATTTTCCACGTCAACAAGGACGTGAAGATCACCTACACCGGCGTCGAGCTGCGGGCCGACGATGACGAACTGGTGTGGCAGCAGGTGTTGGAGTACGCGAAGCGCACCCCTATCGGCGAGCCGATCACCTTCACGTTCTACGAGCTTTGCCAGGACCTGGGCTGGTCGATCAATGGCCGGTATTACACGAAGGCCGAGGAATGCCTGTCGCGCCTACAGGCGACGGCGATGGGCTTCACGTCCGACCGCGTTGGGCACCTGGAATCGGTGTCGCTGCTGCACCGCTTCCGCGTCCTGGACCGTGGCAAGAAAACGTCCCGTTGCCAGGTCCTGATCGACGAGGAAATCGTCGTGCTGTTTGCTGGCGACCACTACACGAAATTCATATGGGAGAAGTACCGCAAGCTGTCGCCGACGGCCCGACGGATGTTCGACTATTTCAGCTCGCACCGGGAGCCGTACCCGCTCAAGCTGGAAACCTTCCGCCTCATGTGCGGATCGGATTCCACCCGCGTGAAGAAGTGGCGCGAGCAGGTCGGCGAAGCCTGCGAAGAGTTGCGAGGCAGCGGCCTGGTGGAACACGCCTGGGTCAATGATGACCTGGTGCATTGCAAACGCTAGGGCCTTGTGGGGTCAGTTCCGGCTGGATCTGCTCTCCCGCTGACGCCGTCCCGGACTGATGGGCTGCCTGTATCGAGTGGTGATTTTGTGCCGAGCTGCCGGTCGGGGAGCTGTTGGCTGGCTGGTGGCAGGATATATTGTGGTGTAAA

>pAg1-hyg-P-T

CAAATTGACGCTTAGACAACTTAATAACACATTGCGGACGTTTTTAATGTACTGGGGTGGTTTTTCTTTTCACCAGTGAGACGGGCAACAGCGGCGCCATTCGCCATTCAGGCTGCGCAACTGTTGGGAAGGGCGATCGGTGCGGGCCTCTTCGCTATTACGCCAGCTGGCGAAAGGGGGATGTGCTGCAAGGCGATTAAGTTGGGTAACGCCAGGGTTTTCCCAGTCACGACGTTGTAAAACGACGGCCAGTGAGCGCGCGTAATACGACTCACTATAGGGCGAATTGGAGCTCCACCGCGGTGGCGGCCGCATGTCAGGAACCTCAACGGCCGGAGTGCGTCGAGCTTGGGCCGAGATTATCGTGAAGCGCCTCCGTCCCTAGCCGGAACCCGTTCTTTTCCATTGCCGCTCATCCACGATCATTTTCGTGGTCCATCGCAGACTTCCGAGGTTAGTGGTGGCCTTGCGAAACAAAGCCGTAGTCAATTCAAACAGCAGGTAATGACTTTCAGGTAAGCGGCAAGCTGCCCGCACCGGTTGGTTGAACTGGTGGCGATGGCACGCCGCAATACGAACCTACCTGAGAGCCAAAGTGATCTTTGCATTGCGGTCTTTCACACAACGAACAGGCCACGCAGGTCGGACAGGCCAAGACCGGACAAAGCTTGGAAATGGGCTGCTTATGAAAGCAGGCCGCCCGATCAGGGAGCTGGCGTCGCCGAGTGTCGCCCGGTGACGGAGGTCCGGCGGCCGTGGCGAGTCTCGACAGGGCGCGCCCACGGAAGGGGGCTATGAAGATACACGGTGACAATGGATTTGTGATGCAATTGCTGACAGTTAATTGGGAGGAAGCCATTGAAAATTGACCCCCCCACGCCGTCAGGAGTGGCCATGGGAAGGCACGCCGCGGAAGTCGCCCACTAACGACAGATGCCAAAACGGAGACGAAAGGGACGGAAGGTCGTCGACATGACCCCACCCCAGCGCCATTTGTGATGCAAGTGCGGCCGAGAGAAGCACCACCCCGCCTTCCAGCTTGGAGGGGTCAAGCATTTTTATTGTCGCTCATGCCACCATAAGACGAAACAAAGTTGGAAGCAAACCCTCTTTTTCTCTCCATCATCCCACAAACCACTCGACCCAGACTCGCTCAATTCTCTTCCATTGCCAACAGCTCACCACTTGACCCTCCAATTTCACATCCAGATCCCATCCATCCCTCAGGTAAGCAAGCTTCCTCTAGCTTCCTCAAAGGTACCCTGAGCTCCCCCAACCTTCGTTTCGCAAGCACCTACCTGGTGGCCCCGCACTGCCTCATCTCGCCTGTATCGGAGCCTACGCGAACCAGCACACTCCTCTCGCGTTTGCTGAGCTGCCCACTGCCGGTCCCGGACTGTCCTGTCTCAGACGACCGACCGACCTGGCTGGAAGCTTAAAAACCCCTTCAGTACCCCGCCTGCCATCCATCCGCTTCCCCCATCATCCATACCTCCGTGTGCTCTCCAACCCCCACGTCCTGTCCTGTGTCATCGTCGTCATCCTTTCTTCTCGACAAGCAGTCTACTCACTCCCATCTCACAGCCAGGATCCATGAAAAAGCCTGAACTCACCGCGACGTCTGTCGAGAAGTTTCTGATCGAAAAGTTCGACAGCGTCTCCGACCTGATGCAGCTCTCGGAGGGCGAAGAATCTCGTGCTTTCAGCTTCGATGTAGGAGGGCGTGGATATGTCCTGCGGGTAAATAGCTGCGCCGATGGTTTCTACAAAGATCGTTATGTTTATCGGCACTTTGCATCGGCCGCGCTCCCGATTCCGGAAGTGCTTGACATTGGGGAATTCAGCGAGAGCCTGACCTATTGCATCTCCCGCCGTGCACAGGGTGTCACGTTGCAAGACCTGCCTGAAACCGAACTGCCCGCTGTTCTGCAGCCGGTCGCGGAGGCCATGGATGCGATCGCTGCGGCCGATCTTAGCCAGACGAGCGGGTTCGGCCCATTCGGACCGCAAGGAATCGGTCAATACACTACATGGCGTGATTTCATATGCGCGATTGCTGATCCCCATGTGTATCACTGGCAAACTGTGATGGACGACACCGTCAGTGCGTCCGTCGCGCAGGCTCTCGATGAGCTGATGCTTTGGGCCGAGGACTGCCCCGAAGTCCGGCACCTCGTGCACGCGGATTTCGGCTCCAACAATGTCCTGACGGACAATGGCCGCATAACAGCGGTCATTGACTGGAGCGAGGCGATGTTCGGGGATTCCCAATACGAGGTCGCCAACATCTTCTTCTGGAGGCCGTGGTTGGCTTGTATGGAGCAGCAGACGCGCTACTTCGAGCGGAGGCATCCGGAGCTTGCAGGATCGCCGCGGCTCCGGGCGTATATGCTCCGCATTGGTCTTGACCAACTCTATCAGAGCTTGGTTGACGGCAATTTCGATGATGCAGCTTGGGCGCAGGGTCGATGCGACGCAATCGTCCGATCCGGAGCCGGGACTGTCGGGCGTACACAAATCGCCCGCAGAAGCGCGGCCGTCTGGACCGATGGCTGTGTAGAAGTACTCGCCGATAGTGGAAACCGACGCCCCAGCACTCGTCCGAGGGCAAAGGAATAGGATATCGTGCAAATTTATAGGCGGCAGTTTGTGACTTATGGAGGGCTTTTGTTAGCCATTGGGAGGAATTGAGTATCTTGGACTCTGAGGAAAAGAGGGTCGGGTACATCTCAACAATGGGAGGGTAAACGAGAACACGAGACACGGTTAGGCAGCCATGAGCACAATATAAATGTGAAAAGCCCAAAGCGAGTGTGTGCCAGTTTGTGTGAATGTGTTCTTGGGAGTGTCATAACTTGCCCACTGTAGTGACCCGCTTGAGCACCAAACTCTTGCGTTTGGAGGAGTTCTTGATGTTATTCATCCTGTTTGTGGTGTAAGTATTACTCTCAACCCAGACATCAGCCATGTTGTGGTGTTACGCTGGTGAGACTTGGGGTATGATGAAGCAAAGGCCGGCAAGAGATCATGATGAGATCAAACTACTTATTTTTAGTTCAAGCAATGCTACTTTATTTGTTTGTGACGTTCACAACCCGGAGAGCAGTCATTCACAACTTTGAGTGCTTTGGCATGATGAATGATTGGATGACATGGGGTGGAGGTTGGGTTGGTCTCGAGGGGGGGCCCGGTACCCAGCTTTTGTTCCCTTTAGTGAGGGTTAATTGCGCGCTTGGCGTAATCATGGTCATAGCTGTTTCCTGTGTGAAATTGTTATCCGCTCACAATTCCACACAACATACGAGCCGGAAGCATAAAGTGTAAAGCCTGGGGTGCCTAATGAGTGAGCTAACTCACATTAATTGCGTTGCGCTCACTGCCCGCTTTCCAGTCGGGAAACCTGTCGTGCCAGCTGGCGAAAGGGGGATGTGCTGCAAGGCGATTAAGTTGGGTAACGCCAGGGTTTTCCCAGTCACGACGTTGTAAAACGACGGCCAGTGAATTCGAGCTCGGTACCAAGGCCCCTGGCGAAAGGGGGATGTGCTGCAAGGCGATTAAGTTGGGTAACGCCAGGGTTTTCCCAGTCACGACGTTGTAAAACGACGGCCAGTGAGCGCGCGTAATACGACTCACTATAGGGCGAATTGGAGCTCCACCGCGGTGGCGGCCGCATGTCAGGAACCTCAACGGCCGGAGTGCGTCGAGCTTGGGCCGAGATTATCGTGAAGCGCCTCCGTCCCTAGCCGGAACCCGTTCTTTTCCATTGCCGCTCATCCACGATCATTTTCGTGGTCCATCGCAGACTTCCGAGGTTAGTGGTGGCCTTGCGAAACAAAGCCGTAGTCAATTCAAACAGCAGGTAATGACTTTCAGGTAAGCGGCAAGCTGCCCGCACCGGTTGGTTGAACTGGTGGCGATGGCACGCCGCAATACGAACCTACCTGAGAGCCAAAGTGATCTTTGCATTGCGGTCTTTCACACAACGAACAGGCCACGCAGGTCGGACAGGCCAAGACCGGACAAAGCTTGGAAATGGGCTGCTTATGAAAGCAGGCCGCCCGATCAGGGAGCTGGCGTCGCCGAGTGTCGCCCGGTGACGGAGGTCCGGCGGCCGTGGCGAGTCTCGACAGGGCGCGCCCACGGAAGGGGGCTATGAAGATACACGGTGACAATGGATTTGTGATGCAATTGCTGACAGTTAATTGGGAGGAAGCCATTGAAAATTGACCCCCCCACGCCGTCAGGAGTGGCCATGGGAAGGCACGCCGCGGAAGTCGCCCACTAACGACAGATGCCAAAACGGAGACGAAAGGGACGGAAGGTCGTCGACATGACCCCACCCCAGCGCCATTTGTGATGCAAGTGCGGCCGAGAGAAGCACCACCCCGCCTTCCAGCTTGGAGGGGTCAAGCATTTTTATTGTCGCTCATGCCACCATAAGACGAAACAAAGTTGGAAGCAAACCCTCTTTTTCTCTCCATCATCCCACAAACCACTCGACCCAGACTCGCTCAATTCTCTTCCATTGCCAACAGCTCACCACTTGACCCTCCAATTTCACATCCAGATCCCATCCATCCCTCAGGTAAGCAAGCTTCCTCTAGCTTCCTCAAAGGTACCCTGAGCTCCCCCAACCTTCGTTTCGCAAGCACCTACCTGGTGGCCCCGCACTGCCTCATCTCGCCTGTATCGGAGCCTACGCGAACCAGCACACTCCTCTCGCGTTTGCTGAGCTGCCCACTGCCGGTCCCGGACTGTCCTGTCTCAGACGACCGACCGACCTGGCTGGAAGCTTAAAAACCCCTTCAGTACCCCGCCTGCCATCCATCCGCTTCCCCCATCATCCATACCTCCGTGTGCTCTCCAACCCCCACGTCCTGTCCTGTGTCATCGTCGTCATCCTTTCTTCTCGACAAGCAGTCTACTCACTCCCATCTCACAGCCAGGATCCCCCGGGCTGCAGGAATTCGATATCGTGCAAATTTATAGGCGGCAGTTTGTGACTTATGGAGGGCTTTTGTTAGCCATTGGGAGGAATTGAGTATCTTGGACTCTGAGGAAAAGAGGGTCGGGTACATCTCAACAATGGGAGGGTAAACGAGAACACGAGACACGGTTAGGCAGCCATGAGCACAATATAAATGTGAAAAGCCCAAAGCGAGTGTGTGCCAGTTTGTGTGAATGTGTTCTTGGGAGTGTCATAACTTGCCCACTGTAGTGACCCGCTTGAGCACCAAACTCTTGCGTTTGGAGGAGTTCTTGATGTTATTCATCCTGTTTGTGGTGTAAGTATTACTCTCAACCCAGACATCAGCCATGTTGTGGTGTTACGCTGGTGAGACTTGGGGTATGATGAAGCAAAGGCCGGCAAGAGATCATGATGAGATCAAACTACTTATTTTTAGTTCAAGCAATGCTACTTTATTTGTTTGTGACGTTCACAACCCGGAGAGCAGTCATTCACAACTTTGAGTGCTTTGGCATGATGAATGATTGGATGACATGGGGTGGAGGTTGGGTTGGTCTCGAGGGGGGGCCCGGTACCCAGCTTTTGTTCCCTTTAGTGAGGGTTAATTGCGCGCTTGGCGTAATCATGGTCATAGCTGTTTCCTGTGTGAAATTGTTATCCGCTCACAATTCCACACAACATACGAGCCGGAAGCATAAAGTGTAAAGCCTGGGGTGCCTAATGAGTGAGCTAACTCACATTAATTGCGTTGCGCTCACTGCCCGCTTTCCAGTCGGGAAACCTGTCGTGCCAGGGGCTGGCCACGGCCGCCTAGGCGCGCAAGGATCCTCTAGATCTAGAGGATCCCCCGACTAGTGCGCGATCGCGGCCGGCCGGCGCGCCGTTTAAACGGATTTAAATTAATTAATGTCGACCTGCAGGCATGCAAGCTTCGTGACTCCCTTAATTCTCCGCTCATGATCAGATTGTCGTTTCCCGCCTTCAGTTTAAACTATCAGTGTTTGACAGGATATATTGGCGGGTAAACCTAAGAGAAAAGAGCGTTTATTAGAATAATCGGATATTTAAAAGGGCGTGAAAAGGTTTATCCGTTCGTCCATTTGTTTGTTCATGCCAACCACAGGGTTCCAGATCCGACGAGCAAGGCAAGACCGAGCGCCTTTGCGACGCTCACCGGGCTGGTTGCCCTCGCCGCTGGGCTGGCGGCCGTCTATGGCCCTGCAAACGCGCCAGAAACGCCGTCGAAGCCGTGTGCGAGACACCGCGGCCGCCGGCGTTGTGGATACCTCGCGGAAAACTTGGCCCTCACTGACAGATGAGGGGCGGACGTTGACACTTGAGGGGCCGACTCACCCGGCGCGGCGTTGACAGATGAGGGGCAGGCTCGATTTCGGCCGGCGACGTGGAGCTGGCCAGCCTCGCAAATCGGCGAAAACGCCTGATTTTACGCGAGTTTCCCACAGATGATGTGGACAAGCCTGGGGATAAGTGCCCTGCGGTATTGACACTTGAGGGGCGCGACTACTGACAGATGAGGGGCGCGATCCTTGACACTTGAGGGGCAGAGTGCTGACAGATGAGGGGCGCACCTATTGACATTTGAGGGGCTGTCCACAGGCAGAAAATCCAGCATTTGCAAGGGTTTCCGCCCGTTTTTCGGCCACCGCTAACCTGTCTTTTAACCTGCTTTTAAACCAATATTTATAAACCTTGTTTTTAACCAGGGCTGCGCCCTGTGCGCGTGACCGCGCACGCCGAAGGGGGGTGCCCCCCCTTCTCGAACCCTCCCGGCCCGCTAACGCGGGCCTCCCATCCCCCCAGGCGTACGCCACTGGAGCACCTCAAAAACACCATCATACACTAAATCAGTAAGTTGGCAGCATCACCCATAATTGTGGTTTCAAAATCGGCTCCGTCGATACTATGTTATACGCCAACTTTGAAAACAACTTTGAAAAAGCTGTTTTCTGGTATTTAAGGTTTTAGAATGCAAGGAACAGTGAATTGGAGTTCGTCTTGTTATAATTAGCTTCTTGGGGTATCTTTAAATACTGTAGAAAAGAGGAAGGAAATAATAAATGGCTAAAATGAGAATATCACCGGAATTGAAAAAACTGATCGAAAAATACCGCTGCGTAAAAGATACGGAAGGAATGTCTCCTGCTAAGGTATATAAGCTGGTGGGAGAAAATGAAAACCTATATTTAAAAATGACGGACAGCCGGTATAAAGGGACCACCTATGATGTGGAACGGGAAAAGGACATGATGCTATGGCTGGAAGGAAAGCTGCCTGTTCCAAAGGTCCTGCACTTTGAACGGCATGATGGCTGGAGCAATCTGCTCATGAGTGAGGCCGATGGCGTCCTTTGCTCGGAAGAGTATGAAGATGAACAAAGCCCTGAAAAGATTATCGAGCTGTATGCGGAGTGCATCAGGCTCTTTCACTCCATCGACATATCGGATTGTCCCTATACGAATAGCTTAGACAGCCGCTTAGCCGAATTGGATTACTTACTGAATAACGATCTGGCCGATGTGGATTGCGAAAACTGGGAAGAAGACACTCCATTTAAAGATCCGCGCGAGCTGTATGATTTTTTAAAGACGGAAAAGCCCGAAGAGGAACTTGTCTTTTCCCACGGCGACCTGGGAGACAGCAACATCTTTGTGAAAGATGGCAAAGTAAGTGGCTTTATTGATCTTGGGAGAAGCGGCAGGGCGGACAAGTGGTATGACATTGCCTTCTGCGTCCGGTCGATCAGGGAGGATATCGGGGAAGAACAGTATGTCGAGCTATTTTTTGACTTACTGGGGATCAAGCCTGATTGGGAGAAAATAAAATATTATATTTTACTGGATGAATTGTTTTAGTACCTAGATGTGGCGCAACGATGCCGGCGACAAGCAGGAGCGCACCGACTTCTTCCGCATCAAGTGTTTTGGCTCTCAGGCCGAGGCCCACGGCAAGTATTTGGGCAAGGGGTCGCTGGTATTCGTGCAGGGCAAGATTCGGAATACCAAGTACGAGAAGGACGGCCAGACGGTCTACGGGACCGACTTCATTGCCGATAAGGTGGATTATCTGGACACCAAGGCACCAGGCGGGTCAAATCAGGAATAAGGGCACATTGCCCCGGCGTGAGTCGGGGCAATCCCGCAAGGAGGGTGAATGAATCGGACGTTTGACCGGAAGGCATACAGGCAAGAACTGATCGACGCGGGGTTTTCCGCCGAGGATGCCGAAACCATCGCAAGCCGCACCGTCATGCGTGCGCCCCGCGAAACCTTCCAGTCCGTCGGCTCGATGGTCCAGCAAGCTACGGCCAAGATCGAGCGCGACAGCGTGCAACTGGCTCCCCCTGCCCTGCCCGCGCCATCGGCCGCCGTGGAGCGTTCGCGTCGTCTCGAACAGGAGGCGGCAGGTTTGGCGAAGTCGATGACCATCGACACGCGAGGAACTATGACGACCAAGAAGCGAAAAACCGCCGGCGAGGACCTGGCAAAACAGGTCAGCGAGGCCAAGCAGGCCGCGTTGCTGAAACACACGAAGCAGCAGATCAAGGAAATGCAGCTTTCCTTGTTCGATATTGCGCCGTGGCCGGACACGATGCGAGCGATGCCAAACGACACGGCCCGCTCTGCCCTGTTCACCACGCGCAACAAGAAAATCCCGCGCGAGGCGCTGCAAAACAAGGTCATTTTCCACGTCAACAAGGACGTGAAGATCACCTACACCGGCGTCGAGCTGCGGGCCGACGATGACGAACTGGTGTGGCAGCAGGTGTTGGAGTACGCGAAGCGCACCCCTATCGGCGAGCCGATCACCTTCACGTTCTACGAGCTTTGCCAGGACCTGGGCTGGTCGATCAATGGCCGGTATTACACGAAGGCCGAGGAATGCCTGTCGCGCCTACAGGCGACGGCGATGGGCTTCACGTCCGACCGCGTTGGGCACCTGGAATCGGTGTCGCTGCTGCACCGCTTCCGCGTCCTGGACCGTGGCAAGAAAACGTCCCGTTGCCAGGTCCTGATCGACGAGGAAATCGTCGTGCTGTTTGCTGGCGACCACTACACGAAATTCATATGGGAGAAGTACCGCAAGCTGTCGCCGACGGCCCGACGGATGTTCGACTATTTCAGCTCGCACCGGGAGCCGTACCCGCTCAAGCTGGAAACCTTCCGCCTCATGTGCGGATCGGATTCCACCCGCGTGAAGAAGTGGCGCGAGCAGGTCGGCGAAGCCTGCGAAGAGTTGCGAGGCAGCGGCCTGGTGGAACACGCCTGGGTCAATGATGACCTGGTGCATTGCAAACGCTAGGGCCTTGTGGGGTCAGTTCCGGCTGGATCTGCTCTCCCGCTGACGCCGTCCCGGACTGATGGGCTGCCTGTATCGAGTGGTGATTTTGTGCCGAGCTGCCGGTCGGGGAGCTGTTGGCTGGCTGGTGGCAGGATATATTGTGGTGTAAA

>pAg-egfp

CAAATTGACGCTTAGACAACTTAATAACACATTGCGGACGTTTTTAATGTACTGGGGTGGTTTTTCTTTTCACCAGTGAGACGGGCAACAGCGGCGCCATTCGCCATTCAGGCTGCGCAACTGTTGGGAAGGGCGATCGGTGCGGGCCTCTTCGCTATTACGCCAGCTGGCGAAAGGGGGATGTGCTGCAAGGCGATTAAGTTGGGTAACGCCAGGGTTTTCCCAGTCACGACGTTGTAAAACGACGGCCAGTGAGCGCGCGTAATACGACTCACTATAGGGCGAATTGGAGCTCCACCGCGGTGGCGGCCGCATGTCAGGAACCTCAACGGCCGGAGTGCGTCGAGCTTGGGCCGAGATTATCGTGAAGCGCCTCCGTCCCTAGCCGGAACCCGTTCTTTTCCATTGCCGCTCATCCACGATCATTTTCGTGGTCCATCGCAGACTTCCGAGGTTAGTGGTGGCCTTGCGAAACAAAGCCGTAGTCAATTCAAACAGCAGGTAATGACTTTCAGGTAAGCGGCAAGCTGCCCGCACCGGTTGGTTGAACTGGTGGCGATGGCACGCCGCAATACGAACCTACCTGAGAGCCAAAGTGATCTTTGCATTGCGGTCTTTCACACAACGAACAGGCCACGCAGGTCGGACAGGCCAAGACCGGACAAAGCTTGGAAATGGGCTGCTTATGAAAGCAGGCCGCCCGATCAGGGAGCTGGCGTCGCCGAGTGTCGCCCGGTGACGGAGGTCCGGCGGCCGTGGCGAGTCTCGACAGGGCGCGCCCACGGAAGGGGGCTATGAAGATACACGGTGACAATGGATTTGTGATGCAATTGCTGACAGTTAATTGGGAGGAAGCCATTGAAAATTGACCCCCCCACGCCGTCAGGAGTGGCCATGGGAAGGCACGCCGCGGAAGTCGCCCACTAACGACAGATGCCAAAACGGAGACGAAAGGGACGGAAGGTCGTCGACATGACCCCACCCCAGCGCCATTTGTGATGCAAGTGCGGCCGAGAGAAGCACCACCCCGCCTTCCAGCTTGGAGGGGTCAAGCATTTTTATTGTCGCTCATGCCACCATAAGACGAAACAAAGTTGGAAGCAAACCCTCTTTTTCTCTCCATCATCCCACAAACCACTCGACCCAGACTCGCTCAATTCTCTTCCATTGCCAACAGCTCACCACTTGACCCTCCAATTTCACATCCAGATCCCATCCATCCCTCAGGTAAGCAAGCTTCCTCTAGCTTCCTCAAAGGTACCCTGAGCTCCCCCAACCTTCGTTTCGCAAGCACCTACCTGGTGGCCCCGCACTGCCTCATCTCGCCTGTATCGGAGCCTACGCGAACCAGCACACTCCTCTCGCGTTTGCTGAGCTGCCCACTGCCGGTCCCGGACTGTCCTGTCTCAGACGACCGACCGACCTGGCTGGAAGCTTAAAAACCCCTTCAGTACCCCGCCTGCCATCCATCCGCTTCCCCCATCATCCATACCTCCGTGTGCTCTCCAACCCCCACGTCCTGTCCTGTGTCATCGTCGTCATCCTTTCTTCTCGACAAGCAGTCTACTCACTCCCATCTCACAGCCAGGATCCATGAAAAAGCCTGAACTCACCGCGACGTCTGTCGAGAAGTTTCTGATCGAAAAGTTCGACAGCGTCTCCGACCTGATGCAGCTCTCGGAGGGCGAAGAATCTCGTGCTTTCAGCTTCGATGTAGGAGGGCGTGGATATGTCCTGCGGGTAAATAGCTGCGCCGATGGTTTCTACAAAGATCGTTATGTTTATCGGCACTTTGCATCGGCCGCGCTCCCGATTCCGGAAGTGCTTGACATTGGGGAATTCAGCGAGAGCCTGACCTATTGCATCTCCCGCCGTGCACAGGGTGTCACGTTGCAAGACCTGCCTGAAACCGAACTGCCCGCTGTTCTGCAGCCGGTCGCGGAGGCCATGGATGCGATCGCTGCGGCCGATCTTAGCCAGACGAGCGGGTTCGGCCCATTCGGACCGCAAGGAATCGGTCAATACACTACATGGCGTGATTTCATATGCGCGATTGCTGATCCCCATGTGTATCACTGGCAAACTGTGATGGACGACACCGTCAGTGCGTCCGTCGCGCAGGCTCTCGATGAGCTGATGCTTTGGGCCGAGGACTGCCCCGAAGTCCGGCACCTCGTGCACGCGGATTTCGGCTCCAACAATGTCCTGACGGACAATGGCCGCATAACAGCGGTCATTGACTGGAGCGAGGCGATGTTCGGGGATTCCCAATACGAGGTCGCCAACATCTTCTTCTGGAGGCCGTGGTTGGCTTGTATGGAGCAGCAGACGCGCTACTTCGAGCGGAGGCATCCGGAGCTTGCAGGATCGCCGCGGCTCCGGGCGTATATGCTCCGCATTGGTCTTGACCAACTCTATCAGAGCTTGGTTGACGGCAATTTCGATGATGCAGCTTGGGCGCAGGGTCGATGCGACGCAATCGTCCGATCCGGAGCCGGGACTGTCGGGCGTACACAAATCGCCCGCAGAAGCGCGGCCGTCTGGACCGATGGCTGTGTAGAAGTACTCGCCGATAGTGGAAACCGACGCCCCAGCACTCGTCCGAGGGCAAAGGAATAGGATATCGTGCAAATTTATAGGCGGCAGTTTGTGACTTATGGAGGGCTTTTGTTAGCCATTGGGAGGAATTGAGTATCTTGGACTCTGAGGAAAAGAGGGTCGGGTACATCTCAACAATGGGAGGGTAAACGAGAACACGAGACACGGTTAGGCAGCCATGAGCACAATATAAATGTGAAAAGCCCAAAGCGAGTGTGTGCCAGTTTGTGTGAATGTGTTCTTGGGAGTGTCATAACTTGCCCACTGTAGTGACCCGCTTGAGCACCAAACTCTTGCGTTTGGAGGAGTTCTTGATGTTATTCATCCTGTTTGTGGTGTAAGTATTACTCTCAACCCAGACATCAGCCATGTTGTGGTGTTACGCTGGTGAGACTTGGGGTATGATGAAGCAAAGGCCGGCAAGAGATCATGATGAGATCAAACTACTTATTTTTAGTTCAAGCAATGCTACTTTATTTGTTTGTGACGTTCACAACCCGGAGAGCAGTCATTCACAACTTTGAGTGCTTTGGCATGATGAATGATTGGATGACATGGGGTGGAGGTTGGGTTGGTCTCGAGGGGGGGCCCGGTACCCAGCTTTTGTTCCCTTTAGTGAGGGTTAATTGCGCGCTTGGCGTAATCATGGTCATAGCTGTTTCCTGTGTGAAATTGTTATCCGCTCACAATTCCACACAACATACGAGCCGGAAGCATAAAGTGTAAAGCCTGGGGTGCCTAATGAGTGAGCTAACTCACATTAATTGCGTTGCGCTCACTGCCCGCTTTCCAGTCGGGAAACCTGTCGTGCCAGCTGGCGAAAGGGGGATGTGCTGCAAGGCGATTAAGTTGGGTAACGCCAGGGTTTTCCCAGTCACGACGTTGTAAAACGACGGCCAGTGAATTCGAGCTCGGTACCAAGGCCCCTGGCGAAAGGGGGATGTGCTGCAAGGCGATTAAGTTGGGTAACGCCAGGGTTTTCCCAGTCACGACGTTGTAAAACGACGGCCAGTGAGCGCGCGTAATACGACTCACTATAGGGCGAATTGGAGCTCCACCGCGGTGGCGGCCGCATGTCAGGAACCTCAACGGCCGGAGTGCGTCGAGCTTGGGCCGAGATTATCGTGAAGCGCCTCCGTCCCTAGCCGGAACCCGTTCTTTTCCATTGCCGCTCATCCACGATCATTTTCGTGGTCCATCGCAGACTTCCGAGGTTAGTGGTGGCCTTGCGAAACAAAGCCGTAGTCAATTCAAACAGCAGGTAATGACTTTCAGGTAAGCGGCAAGCTGCCCGCACCGGTTGGTTGAACTGGTGGCGATGGCACGCCGCAATACGAACCTACCTGAGAGCCAAAGTGATCTTTGCATTGCGGTCTTTCACACAACGAACAGGCCACGCAGGTCGGACAGGCCAAGACCGGACAAAGCTTGGAAATGGGCTGCTTATGAAAGCAGGCCGCCCGATCAGGGAGCTGGCGTCGCCGAGTGTCGCCCGGTGACGGAGGTCCGGCGGCCGTGGCGAGTCTCGACAGGGCGCGCCCACGGAAGGGGGCTATGAAGATACACGGTGACAATGGATTTGTGATGCAATTGCTGACAGTTAATTGGGAGGAAGCCATTGAAAATTGACCCCCCCACGCCGTCAGGAGTGGCCATGGGAAGGCACGCCGCGGAAGTCGCCCACTAACGACAGATGCCAAAACGGAGACGAAAGGGACGGAAGGTCGTCGACATGACCCCACCCCAGCGCCATTTGTGATGCAAGTGCGGCCGAGAGAAGCACCACCCCGCCTTCCAGCTTGGAGGGGTCAAGCATTTTTATTGTCGCTCATGCCACCATAAGACGAAACAAAGTTGGAAGCAAACCCTCTTTTTCTCTCCATCATCCCACAAACCACTCGACCCAGACTCGCTCAATTCTCTTCCATTGCCAACAGCTCACCACTTGACCCTCCAATTTCACATCCAGATCCCATCCATCCCTCAGGTAAGCAAGCTTCCTCTAGCTTCCTCAAAGGTACCCTGAGCTCCCCCAACCTTCGTTTCGCAAGCACCTACCTGGTGGCCCCGCACTGCCTCATCTCGCCTGTATCGGAGCCTACGCGAACCAGCACACTCCTCTCGCGTTTGCTGAGCTGCCCACTGCCGGTCCCGGACTGTCCTGTCTCAGACGACCGACCGACCTGGCTGGAAGCTTAAAAACCCCTTCAGTACCCCGCCTGCCATCCATCCGCTTCCCCCATCATCCATACCTCCGTGTGCTCTCCAACCCCCACGTCCTGTCCTGTGTCATCGTCGTCATCCTTTCTTCTCGACAAGCAGTCTACTCACTCCCATCTCACAGCCAGGATCCCCCATCATGGTGAGCAAGGGCGAGGAGCTGTTCACCGGGGTGGTGCCCATCCTGGTCGAGCTGGACGGCGACGTAAACGGCCACAAGTTCAGCGTGTCCGGCGAGGGCGAGGGCGATGCCACCTACGGCAAGCTGACCCTGAAGTTCATCTGCACCACCGGCAAGCTGCCCGTGCCCTGGCCCACCCTCGTGACCACCCTGACCTACGGCGTGCAGTGCTTCAGCCGCTACCCCGACCACATGAAGCAGCACGACTTCTTCAAGTCCGCCATGCCCGAAGGCTACGTCCAGGAGCGCACCATCTTCTTCAAGGACGACGGCAACTACAAGACCCGCGCCGAGGTGAAGTTCGAGGGCGACACCCTGGTGAACCGCATCGAGCTGAAGGGCATCGACTTCAAGGAGGACGGCAACATCCTGGGGCACAAGCTGGAGTACAACTACAACAGCCACAACGTCTATATCATGGCCGACAAGCAGAAGAACGGCATCAAGGTGAACTTCAAGATCCGCCACAACATCGAGGACGGCAGCGTGCAGCTCGCCGACCACTACCAGCAGAACACCCCCATCGGCGACGGCCCCGTGCTGCTGCCCGACAACCACTACCTGAGCACCCAGTCCGCCCTGAGCAAAGACCCCAACGAGAAGCGCGATCACATGGTCCTGCTGGAGTTCGTGACCGCCGCCGGGATCACTCTCGGCATGGACGAGCTGTACAAGTGATGGGCTGCAGGAATTCGATATCGTGCAAATTTATAGGCGGCAGTTTGTGACTTATGGAGGGCTTTTGTTAGCCATTGGGAGGAATTGAGTATCTTGGACTCTGAGGAAAAGAGGGTCGGGTACATCTCAACAATGGGAGGGTAAACGAGAACACGAGACACGGTTAGGCAGCCATGAGCACAATATAAATGTGAAAAGCCCAAAGCGAGTGTGTGCCAGTTTGTGTGAATGTGTTCTTGGGAGTGTCATAACTTGCCCACTGTAGTGACCCGCTTGAGCACCAAACTCTTGCGTTTGGAGGAGTTCTTGATGTTATTCATCCTGTTTGTGGTGTAAGTATTACTCTCAACCCAGACATCAGCCATGTTGTGGTGTTACGCTGGTGAGACTTGGGGTATGATGAAGCAAAGGCCGGCAAGAGATCATGATGAGATCAAACTACTTATTTTTAGTTCAAGCAATGCTACTTTATTTGTTTGTGACGTTCACAACCCGGAGAGCAGTCATTCACAACTTTGAGTGCTTTGGCATGATGAATGATTGGATGACATGGGGTGGAGGTTGGGTTGGTCTCGAGGGGGGGCCCGGTACCCAGCTTTTGTTCCCTTTAGTGAGGGTTAATTGCGCGCTTGGCGTAATCATGGTCATAGCTGTTTCCTGTGTGAAATTGTTATCCGCTCACAATTCCACACAACATACGAGCCGGAAGCATAAAGTGTAAAGCCTGGGGTGCCTAATGAGTGAGCTAACTCACATTAATTGCGTTGCGCTCACTGCCCGCTTTCCAGTCGGGAAACCTGTCGTGCCAGGGGCTGGCCACGGCCGCCTAGGCGCGCAAGGATCCTCTAGATCTAGAGGATCCCCCGACTAGTGCGCGATCGCGGCCGGCCGGCGCGCCGTTTAAACGGATTTAAATTAATTAATGTCGACCTGCAGGCATGCAAGCTTCGTGACTCCCTTAATTCTCCGCTCATGATCAGATTGTCGTTTCCCGCCTTCAGTTTAAACTATCAGTGTTTGACAGGATATATTGGCGGGTAAACCTAAGAGAAAAGAGCGTTTATTAGAATAATCGGATATTTAAAAGGGCGTGAAAAGGTTTATCCGTTCGTCCATTTGTTTGTTCATGCCAACCACAGGGTTCCAGATCCGACGAGCAAGGCAAGACCGAGCGCCTTTGCGACGCTCACCGGGCTGGTTGCCCTCGCCGCTGGGCTGGCGGCCGTCTATGGCCCTGCAAACGCGCCAGAAACGCCGTCGAAGCCGTGTGCGAGACACCGCGGCCGCCGGCGTTGTGGATACCTCGCGGAAAACTTGGCCCTCACTGACAGATGAGGGGCGGACGTTGACACTTGAGGGGCCGACTCACCCGGCGCGGCGTTGACAGATGAGGGGCAGGCTCGATTTCGGCCGGCGACGTGGAGCTGGCCAGCCTCGCAAATCGGCGAAAACGCCTGATTTTACGCGAGTTTCCCACAGATGATGTGGACAAGCCTGGGGATAAGTGCCCTGCGGTATTGACACTTGAGGGGCGCGACTACTGACAGATGAGGGGCGCGATCCTTGACACTTGAGGGGCAGAGTGCTGACAGATGAGGGGCGCACCTATTGACATTTGAGGGGCTGTCCACAGGCAGAAAATCCAGCATTTGCAAGGGTTTCCGCCCGTTTTTCGGCCACCGCTAACCTGTCTTTTAACCTGCTTTTAAACCAATATTTATAAACCTTGTTTTTAACCAGGGCTGCGCCCTGTGCGCGTGACCGCGCACGCCGAAGGGGGGTGCCCCCCCTTCTCGAACCCTCCCGGCCCGCTAACGCGGGCCTCCCATCCCCCCAGGCGTACGCCACTGGAGCACCTCAAAAACACCATCATACACTAAATCAGTAAGTTGGCAGCATCACCCATAATTGTGGTTTCAAAATCGGCTCCGTCGATACTATGTTATACGCCAACTTTGAAAACAACTTTGAAAAAGCTGTTTTCTGGTATTTAAGGTTTTAGAATGCAAGGAACAGTGAATTGGAGTTCGTCTTGTTATAATTAGCTTCTTGGGGTATCTTTAAATACTGTAGAAAAGAGGAAGGAAATAATAAATGGCTAAAATGAGAATATCACCGGAATTGAAAAAACTGATCGAAAAATACCGCTGCGTAAAAGATACGGAAGGAATGTCTCCTGCTAAGGTATATAAGCTGGTGGGAGAAAATGAAAACCTATATTTAAAAATGACGGACAGCCGGTATAAAGGGACCACCTATGATGTGGAACGGGAAAAGGACATGATGCTATGGCTGGAAGGAAAGCTGCCTGTTCCAAAGGTCCTGCACTTTGAACGGCATGATGGCTGGAGCAATCTGCTCATGAGTGAGGCCGATGGCGTCCTTTGCTCGGAAGAGTATGAAGATGAACAAAGCCCTGAAAAGATTATCGAGCTGTATGCGGAGTGCATCAGGCTCTTTCACTCCATCGACATATCGGATTGTCCCTATACGAATAGCTTAGACAGCCGCTTAGCCGAATTGGATTACTTACTGAATAACGATCTGGCCGATGTGGATTGCGAAAACTGGGAAGAAGACACTCCATTTAAAGATCCGCGCGAGCTGTATGATTTTTTAAAGACGGAAAAGCCCGAAGAGGAACTTGTCTTTTCCCACGGCGACCTGGGAGACAGCAACATCTTTGTGAAAGATGGCAAAGTAAGTGGCTTTATTGATCTTGGGAGAAGCGGCAGGGCGGACAAGTGGTATGACATTGCCTTCTGCGTCCGGTCGATCAGGGAGGATATCGGGGAAGAACAGTATGTCGAGCTATTTTTTGACTTACTGGGGATCAAGCCTGATTGGGAGAAAATAAAATATTATATTTTACTGGATGAATTGTTTTAGTACCTAGATGTGGCGCAACGATGCCGGCGACAAGCAGGAGCGCACCGACTTCTTCCGCATCAAGTGTTTTGGCTCTCAGGCCGAGGCCCACGGCAAGTATTTGGGCAAGGGGTCGCTGGTATTCGTGCAGGGCAAGATTCGGAATACCAAGTACGAGAAGGACGGCCAGACGGTCTACGGGACCGACTTCATTGCCGATAAGGTGGATTATCTGGACACCAAGGCACCAGGCGGGTCAAATCAGGAATAAGGGCACATTGCCCCGGCGTGAGTCGGGGCAATCCCGCAAGGAGGGTGAATGAATCGGACGTTTGACCGGAAGGCATACAGGCAAGAACTGATCGACGCGGGGTTTTCCGCCGAGGATGCCGAAACCATCGCAAGCCGCACCGTCATGCGTGCGCCCCGCGAAACCTTCCAGTCCGTCGGCTCGATGGTCCAGCAAGCTACGGCCAAGATCGAGCGCGACAGCGTGCAACTGGCTCCCCCTGCCCTGCCCGCGCCATCGGCCGCCGTGGAGCGTTCGCGTCGTCTCGAACAGGAGGCGGCAGGTTTGGCGAAGTCGATGACCATCGACACGCGAGGAACTATGACGACCAAGAAGCGAAAAACCGCCGGCGAGGACCTGGCAAAACAGGTCAGCGAGGCCAAGCAGGCCGCGTTGCTGAAACACACGAAGCAGCAGATCAAGGAAATGCAGCTTTCCTTGTTCGATATTGCGCCGTGGCCGGACACGATGCGAGCGATGCCAAACGACACGGCCCGCTCTGCCCTGTTCACCACGCGCAACAAGAAAATCCCGCGCGAGGCGCTGCAAAACAAGGTCATTTTCCACGTCAACAAGGACGTGAAGATCACCTACACCGGCGTCGAGCTGCGGGCCGACGATGACGAACTGGTGTGGCAGCAGGTGTTGGAGTACGCGAAGCGCACCCCTATCGGCGAGCCGATCACCTTCACGTTCTACGAGCTTTGCCAGGACCTGGGCTGGTCGATCAATGGCCGGTATTACACGAAGGCCGAGGAATGCCTGTCGCGCCTACAGGCGACGGCGATGGGCTTCACGTCCGACCGCGTTGGGCACCTGGAATCGGTGTCGCTGCTGCACCGCTTCCGCGTCCTGGACCGTGGCAAGAAAACGTCCCGTTGCCAGGTCCTGATCGACGAGGAAATCGTCGTGCTGTTTGCTGGCGACCACTACACGAAATTCATATGGGAGAAGTACCGCAAGCTGTCGCCGACGGCCCGACGGATGTTCGACTATTTCAGCTCGCACCGGGAGCCGTACCCGCTCAAGCTGGAAACCTTCCGCCTCATGTGCGGATCGGATTCCACCCGCGTGAAGAAGTGGCGCGAGCAGGTCGGCGAAGCCTGCGAAGAGTTGCGAGGCAGCGGCCTGGTGGAACACGCCTGGGTCAATGATGACCTGGTGCATTGCAAACGCTAGGGCCTTGTGGGGTCAGTTCCGGCTGGATCTGCTCTCCCGCTGACGCCGTCCCGGACTGATGGGCTGCCTGTATCGAGTGGTGATTTTGTGCCGAGCTGCCGGTCGGGGAGCTGTTGGCTGGCTGGTGGCAGGATATATTGTGGTGTAAA
